# Oral infection by *Pseudomonas aeruginosa* in patient with chronic kidney disease - a case report

**DOI:** 10.1590/1678-4685-JBN-3812

**Published:** 2018-04-19

**Authors:** Luana Carneiro Diniz Souza, Fernanda Ferreira Lopes, Eider Guimarães Bastos, Cláudia Maria Coelho Alves

**Affiliations:** 1 Universidade Federal do Maranhão, Programa de Pós-Graduação em Odontologia, São Luís, MA, Brasil.; 2 Universidade Federal do Maranhão, Departamento de Odontologia, São Luís, MA, Brasil.

**Keywords:** Pseudomonas aeruginosa, Shock, Septic, Mouth, Pseudomonas aeruginosa, Choque Séptico, Boca

## Abstract

Chronic renal patients are more susceptible to hospital complications and infections such as urinary tract infections, peritonitis, surgery infections, and bacteremia, which are often caused by *Pseudomonas aeruginosa*. A case of a HIV-positive girl with chronic kidney disease and with serious oral lesions due to *P. aeruginosa* septic shock is presented. The patient showed necrotic lesions in the oral mucosa, pathological tooth mobility, bone loss, and hematogenous osteomyelitis in the maxilla. The patient was submitted to systemic antibiotic therapy based on screening culture and treatment of bone lesions by eliminating the causal agent and restoring health conditions. This case report is extremely important for health professionals, since the oral cavity can be affected by this pathogen or serve as a colonization site.

## RESUMO

Pacientes renais crônicos são mais suscetíveis a complicações e infecções hospitalares, tais como infecções do trato urinário, peritonite, infecções cirúrgicas e bacteremia, muitas vezes causadas por *Pseudomonas aeruginosa*. O presente relato apresenta o caso de uma menina HIV-positiva com doença renal crônica e lesões orais graves devido a choque séptico por *P. aeruginosa*. A paciente apresentava lesões necróticas na mucosa oral, mobilidade dentária patológica, perda óssea e osteomielite hematogênica maxilar. A paciente foi submetida a antibioticoterapia sistêmica com base na cultura de triagem e tratamento das lesões ósseas por meio da eliminação do agente causal e restauração das condições de saúde. Este relato de caso é extremamente importante para os profissionais da saúde, uma vez que a cavidade oral pode ser afetada por este patógeno ou servir como local de colonização.

## INTRODUCTION

Many pediatric patients develop chronic kidney disease (CKD) secondary to congenital abnormalities in the urinary system.^1^ Chronic renal patients are more susceptible to hospital complications^2^ and infections by *Pseudomonas aeruginosa* such as urinary tract infections, peritonitis, surgery infections, and bacteremia. Bacteremia and sepsis result in high mortality rates, especially in patients with cancer, CKD, diabetes, and cardiopulmonary disorders.^3^

*Pseudomonas aeruginosa* is a gram-negative, aerobic, non-sporulating, non-glucose fermentor, and mobile bacilli due to the presence of a polar flagellum.^3^^,^ 4 It is often associated with infections in immunosuppressed patients admitted to intensive care units (ICU), due to the constant exposure to invasive procedures such as catheters, probes, instruments, and devices for mechanical ventilation.^3^

This bacterial species has been considered an opportunistic pathogen, since it is rarely associated with infection in immunocompetent individuals.^3^^,^ 5 *P. aeruginosa* is also associated with mandibular osteomyelitis by intraosseous dissemination^6^ and to necrotic lesions of the oral mucosa of immunosuppressed patients.^7^

The oral cavity can serve as a reservoir for *P. aeruginosa* and, in debilitated patients such as elderly and immunocompromised patients, infections with the pathogen are a major health problem.^8^ Thus, the aim of this study was to report the case of a child with CKD undergoing peritoneal dialysis with severe oral lesions caused by infection with *P. aeruginosa*.

## CASE REPORT

A six-year-old girl with CKD (single kidney, congenital) undergoing peritoneal dialysis presented with fever and a lesion suggestive of linear erythema throughout the marginal gingiva. The patient reported pain in the gingival region and difficulty eating and drinking.

**Figure 1:**
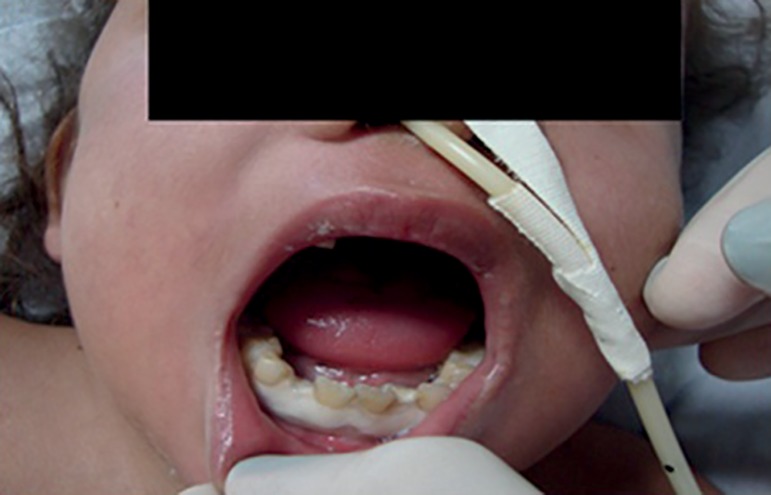
First week of ICU admission (marginal and attached gingiva necrosis).

The initial diagnosis was pneumonia with febrile neutropenia and hypoalbuminemia. Due to the clinical condition, the patient was admitted to pediatric ICU. After a few days of hospitalization, the entire length of the marginal and attached gingiva, both on the buccal and palatal sides, showed white coloration suggestive of necrosis (Figure 1). No dental caries was observed.

A fragment of the marginal gingiva was collected for screening culture, in which the presence of *P. aeruginosa* was detected. As the pathogen was also detected in blood and nasal secretion cultures, the case was diagnosed as septic shock.

**Figure 2:**
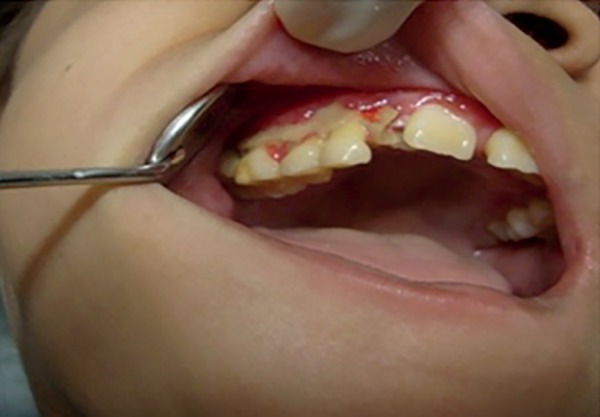
Aspect of the upper arch after the first cycle of systemic antibiotic therapy (elimination of gingival necrosis)

Systemic antibiotic therapy was started (vancomycin, ampicillin-sulbactam, amikacin, piperacillin-tazobactam and polymyxin B) and after a period of hospitalization, a gradual elimination of necrotic gingival mucosa was observed. A hard tissue with bone appearance was present in the remaining normal gingiva, but presented as a fragile structure (Figure 2).

**Figure 3:**
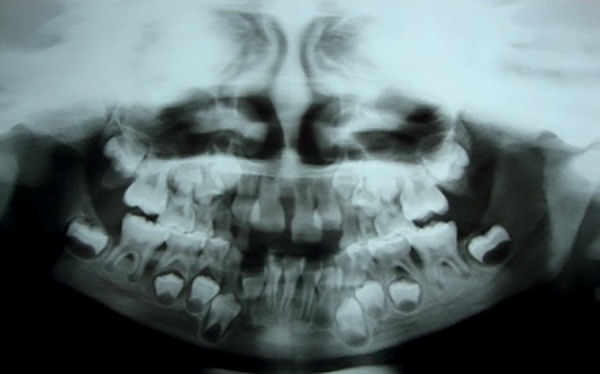
Panoramic radiograph showing bone loss, especially between permanent incisors and molars.

Pathological tooth mobility was present as well as bone loss especially between the first permanent molars and incisors both in the upper and lower jaws (Figure 3). There was clinical insertion loss but without periodontal pockets (Figure 2).

**Figure 4:**
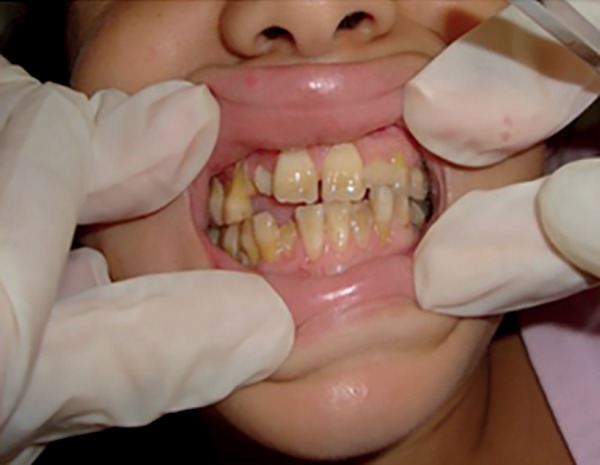
Appearance after supragingival scraping, use of 0.12% chlorhexidine and second cycle of systemic antibiotic therapy.

**Figure 5:**
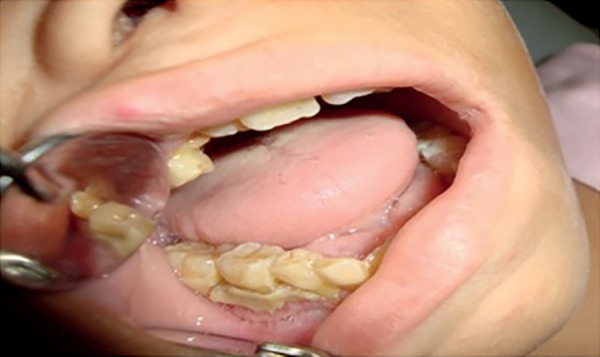
Bone alterations.

Supragingival scraping sessions and application of 0.12% chlorhexidine were performed during the ICU stay. After biopsy of the gingival tissue and the exposed hard tissue, the presence of inflammatory hyperplastic gingivitis was confirmed and the hard tissue was confirmed as bone tissue (Figures 4 and 5). The bone tissue was submitted to culture, which also showed the presence of *P. aeruginosa*. Systemic antibiotic therapy based on levofloxacin was established for another fifteen days.

**Figure 6:**
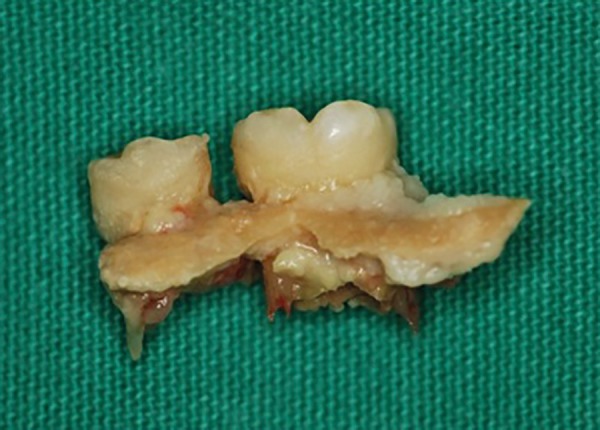
Bone lesion and loose teeth surgically removed.

Bone lesions and some deciduous teeth with high mobility were surgically removed, under antibiotic prophylaxis; antibiotic therapy was maintained postoperatively for seven days. The collected material was sent for histopathology and culture examinations (Figure 6).

Histopathology showed a chronic inflammatory process in necrotic tissue, resulting in the diagnosis of chronic osteomyelitis. *P. aeruginosa* was not detected in culture examination.

**Figure 7:**
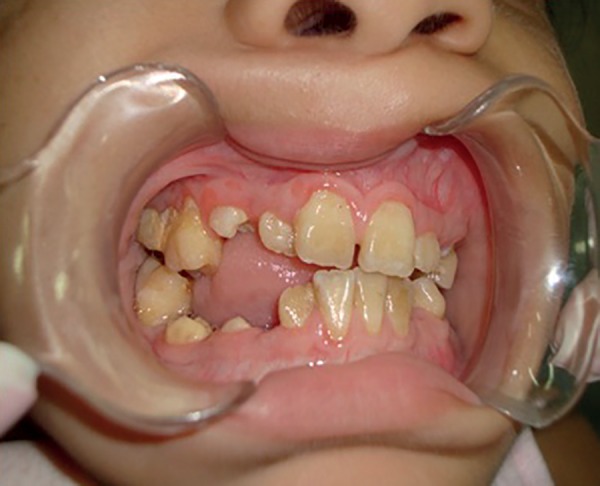
Appearance 15 days after surgery.

Fifteen days later, the patient had significant oral health improvement (Figure 7). After the six-month treatment period, the human immunodeficiency virus (HIV) was detected, although the patient had negative serology in previous tests.

## DISCUSSION

While most cases of maxillary osteomyelitis are secondary to dental infections, hematogenous osteomyelitis can also occur, being more common in pediatric patients.^6^ The pathogens most commonly related to this condition are *Staphylococcus*, *Peptostreptococcus*, and *P. aeruginosa*, among others.^9^ The case presented is in accordance with this information as the patient had maxillary osteomyelitis due to the hematogenous dissemination of *P. aeruginosa*.

Debilitated patients in ICU or in long-term catheter use can develop osteomyelitis caused by *Pseudomonas.* 6 Infections by this bacterium are frequent and of great importance in HIV-infected patients due to the tendency for relapse and easy dissemination. The broad spectrum of clinical features, possible serious complications, and the unpredictable antimicrobial susceptibility make the infection to be of difficult treatment.^10^ During treatment, the patient was probably in the “immunological window” period, as she had negative serology in previous tests. The HIV infection explains the low immunity and the high degree of bone destruction in the maxilla.

Focal necrotizing lesions have been found in the oral mucosa of HIV-positive patients, which are distinct from periodontal disease patterns and associated with the presence of *P. aeruginosa*. Healing of the lesions was observed after systemic antibiotic therapy with concomitant disappearance of *the infection* in subsequent culture examinations.^7^ The same was observed in the present case.

In most *P. aeruginosa* infections, signs and symptoms are non-specific and related to the affected organ. The pathogen penetrates the affected skin or mucosa, invades locally and may enter the blood stream causing septicemia.^11^ In the present case, the bacteria settled in the oral cavity, blood, and lungs.

Because of the high morbidity and mortality in immunocompromised patients, Barasch *et al*.^7^ suggest including *P. aeruginosa* infection in the differential diagnosis of oral necrotic lesions in the absence of necrotizing periodontal disease; the authors emphasize the importance of culture examinations to select the appropriate therapy to the specific pathogen. Similar procedures were carried out in this case as necrotic lesions in marginal and inserted gingiva induced by *P. aeruginosa* were treated with systemic antibiotics based on the susceptibility profile.

Due to the systemic conditions of the patient, who was HIV-positive and had CKD, the immune system was seriously affected, favoring the infection with *P. aeruginosa*. Antibiotic treatment eliminated the causal agent, restoring health conditions. This report is extremely important for health professionals, since the oral cavity can be affected by this pathogen or serve as site for its colonization.
